# Dysphagia Risk and Its Association with Nutritional Status in Multiple Sclerosis: A Preliminary Study

**DOI:** 10.3390/nu18091315

**Published:** 2026-04-22

**Authors:** Nicole Vanessa Franchina Vergel, Jorge Molina-López, Elena Planells

**Affiliations:** 1 Doctoral Programme in Nutrition and Food Science, Department of Physiology, Faculty of Pharmacy, Institute of Nutrition and Food Technology “José Mataix”, University of Granada, 18071 Granada, Spain; elenamp@ugr.es; 2 Faculty of Education, Psychology and Sports Sciences, COIDESO, University of Huelva, 21007 Huelva, Spain; jorge.molina@ddi.uhu.es

**Keywords:** swallowing disorders, nutritional assessment, bioelectrical impedance analysis, body composition, speech and language therapist

## Abstract

**Background/Objectives**: Multiple sclerosis (MS) is a chronic, demyelinating and neurodegenerative disease frequently associated with dysphagia, nutritional imbalances, and alterations in body composition. This study aims to describe the anthropometric profile and body composition in people with MS, estimate the risk and type of dysphagia, analyse dietary intake and habits, and evaluate the evolution of these parameters over six months. **Methods**: This descriptive analytical longitudinal study included 30 patients with MS (20 women, 10 men), with a median age of 53.3 years at baseline and 54.0 years at final assessment. The prevalence of dysphagia risk was determined, dietary patterns and body composition were characterised, and their interactions were explored through two assessments conducted six months apart. **Results**: Overall, 90% of the sample had relapsing–remitting MS (RRMS). At both the initial and final assessments, the median BMI was above 25 kg/m^2^ and a high prevalence of dysphagia risk (63.3% and 76.7%), particularly for liquids. Frequent inadequacies were observed in the intake of certain macronutrients and micronutrients, including energy, fibre, potassium and magnesium. Likewise, the analysis by food groups revealed low adherence to recommendations, particularly for fruits, cereals, legumes, fish and lean meats. No significant differences were detected between the two time points. **Conclusions**: Dysphagia, dietary intake, habits, and body composition are interconnected dimensions in MS; systematically integrating nutritional assessment and dysphagia screening into clinical practice would contribute to a more comprehensive management and to improvements in swallowing disorders and nutritional status in people with MS.

## 1. Introduction

Globally, the WHO estimates the prevalence of multiple sclerosis (MS) to be around 1.8 million people, with nearly 550,000 cases in Spain [1]. It is estimated that between 21 and 23 million people in Spain have neurological diseases; within this group, MS has a significant presence [1]. MS is a neurodegenerative, inflammatory, chronic, and autoimmune disease of the central nervous system, characterised by the demyelination of the myelin sheaths of neurons, leading to permanent deterioration or damage to nerve fibres, with potential permanent neurological impairment [2,3]. It has an unknown aetiology and is multifactorial [2]; it usually manifests between the ages of 20 and 40 and has a higher incidence in women [2,4]. Among the determining factors involved are genetic and environmental factors, including geographical latitude, family history, vitamin D deficiency, obesity, smoking, and dietary patterns [2,3,4,5].

MS comprises several clinical phenotypes that differ in their course and progression [2]. The most common form is relapsing–remitting MS (RRMS), which accounts for approximately 85% of initial presentations and is characterised by episodes of neurological dysfunction followed by partial or complete recovery. Primary progressive MS (PPMS), present in around 10–20% of cases, shows a steady accumulation of disability from onset without clearly defined relapses. Secondary progressive MS (SPMS) develops after an initial relapsing–remitting phase and is defined by a gradual, continuous worsening of neurological function over time. A less frequent phenotype, progressive–relapsing MS, is characterised by a progressive course from onset with superimposed acute relapse. The clinical signs of MS are heterogeneous and depend on the location and severity of the lesions, which may compromise motor, sensory, visual pathways, and brainstem structures [2]. These alterations result in a wide range of symptoms, including fatigue, motor and sensory deficits, visual manifestations, cerebellar and brainstem symptoms, as well as sphincter and cognitive dysfunctions [2,3,6]. The combination of certain symptoms such as motor, sensory, cerebellar or brainstem deficits could lead to the onset of dysphagia, i.e., alterations in the swallowing process [7,8,9].

Dysphagia is an increasingly prevalent complication in MS, which can manifest at any stage of the disease, although its incidence tends to increase as the disease progresses [6,9]. It is estimated to affect approximately 30–40% of patients, with even higher figures in advanced stages [6,9]. This disorder poses a significant clinical risk, as it compromises the safety and efficacy of the swallowing process, promoting the occurrence of events such as food penetration and aspiration, as well as nutritional disorders such as malnutrition, undernourishment, and dehydration [8,10]. These consequences have a negative impact on the quality of life of those affected and are associated with an increase in morbidity and mortality [11,12].

The close association between dysphagia and nutritional disorders reinforces the need to integrate nutritional status assessment and treatment into the multidisciplinary management of MS [13]. In recent years, there has been growing interest in the role of diet in the progression of the disease; recent studies indicate that a balanced diet, combined with physical activity, can have favourable effects on the clinical course of MS [14,15]. Several studies have attempted to outline the most appropriate dietary pattern for this population, although the evidence focuses predominantly on the intake of certain macro- and micronutrients. Foods rich in antioxidants stand out for their potential to modulate inflammation, reduce oxidative stress, and contribute to protection against demyelination; calcium and vitamin D for their role in preserving bone health and immunomodulation; and certain minerals and vitamins (such as vitamin A) for their possible contribution to reducing fatigue [14,16,17,18]. Furthermore, growing interest in nutrition in MS has revealed an anthropometric profile characterised by a higher prevalence of overweight and above-normal body mass index (BMI) values [16,19]. These aspects could be influenced by factors intrinsic to the disease, including motor impairment, which reduces the ability to engage in physical activity and encourages more sedentary behaviours [20]. Moreover, the use of certain drugs can induce pro-inflammatory and metabolic changes that contribute to weight gain and anthropometric alterations [19].

Currently, two seemingly contradictory nutritional realities coexist in MS: on the one hand, a high prevalence of overweight and obesity linked to a sedentary lifestyle secondary to motor impairment and the metabolic effects of certain treatments [21,22]; on the other hand, the presence of dysphagia in advanced stages, which can lead to malnutrition, undernourishment, and dehydration [8,12,23]. This duality highlights the need for regular, personalised nutritional assessments that identify both excess weight and the risk of nutritional deficiency due to swallowing disorders. A multidisciplinary approach is therefore essential to optimise nutritional status, minimise complications, and improve the quality of life of people with MS. Based on the above, the main objective of this study is to describe the prevalence and characteristics of dysphagia risk and nutritional status in people with MS. To support this aim, the following secondary objectives were established: (I) identify the anthropometric profile and body composition of individuals with MS; (II) determine the risk of dysphagia and the type of dysphagia; (III) examine food intake and dietary habits; and (IV) analyse whether the parameters evaluated change over a period of 6 months.

## 2. Materials and Methods

### 2.1. Design

Descriptive analytical study with a longitudinal design in patients with a confirmed diagnosis of MS. The research was conducted at the University of Granada between January 2022 and December 2024. The project was approved by the Biomedical Research Ethics Committee of the Province of Granada, registration number 0972-N-23, and was carried out in accordance with the ethical guidelines established in the Declaration of Helsinki (2024 revision).

### 2.2. Participants

The study was proposed to a group of people diagnosed with MS and, after the selection process, 30 subjects residing in the province of Granada were included. Access to the study population was facilitated by the Hospital Pharmacy Service of the Virgen de las Nieves University Hospital and the Association of People Affected by Neurodegenerative Diseases of Granada (GAEN). The study included 30 participants (20 women and 10 men), who agreed to participate on an ongoing basis and signed the informed consent form in accordance with current ethical regulations. The sample showed a female prevalence of 66.7% and the age ranges were 45.3–59.8 years. The inclusion criteria were as follows: (1) participants of both sexes with a confirmed diagnosis of MS; (2) absence of recognised dysphagia; (3) age ≥ 18 years; (4) absence of pathologies that compromised swallowing abilities; and (5) willingness and availability to actively collaborate in the study, having given informed consent for participation. The diagnosis of MS was confirmed by the Neurology Service of the hospitals in the province of Granada (Andalusian Health System), to which the study subjects belonged. This service applied the McDonald criteria in 2017 [24,25], which require evidence of episodes spread out over time and space, the exclusion of alternative diagnoses, and confirmation through additional tests, including magnetic resonance imaging.

### 2.3. Instruments

Various assessment tools were used in the initial and final evaluations, organised into two blocks: nutritional and speech therapy.

#### 2.3.1. The Nutritional Assessment

For the anthropometric and body-composition assessment, a NUTRILAB™ multifrequency bioimpedance body composition analyser (Akern Srl, Florence, Italy) was used. In turn, dietary intake and food-consumption habits were collected using two complementary tools: a 72 h retrospective food consumption recall (over 3 consecutive days) and a food frequency questionnaire (FFQ), both applied according to standardised protocols for the collection of anthropometric and dietary data.

- The NUTRILAB™ analyser was used to obtain body composition data and anthropometric measurements from the sample. The data were analysed using Bodygram^®^ HBO software (Akern Srl, Florence, Italy), which integrates tools for assessing body composition, hydration (Hydragram^®^, Akern Srl, Florence, Italy), and nutritional status (Nutrigram^®^, Akern Srl, Florence, Italy) using BIVA vector analysis [26]. Hydragram^®^ classified hydration according to impedance vector displacement, defining normal hydration as between 72.7–73.3%. Values outside this range indicated overhydration or dehydration. Nutrigram^®^ estimated nutritional status based on urinary creatinine excretion (UCr/24 h), normalised by height and derived from body cell mass obtained by bioelectrical impedance analysis (BIA). Values > 520 mg/24 h/htm were considered normal; lower figures indicated protein–energy malnutrition [26]. The parameters in this study included BMI, fat mass (FM), fat-free mass (FFM), basal metabolic rate (BMR), Nutrigram, and Hydragram. All measurements were taken by the same researcher and were performed after complying with pre-assessment instructions such as prior urination, absence of intense physical activity in the preceding hours, fasting from food and drink, and removal of metal objects that could interfere with the measurement.
- Food consumption records were obtained through a recall in which participants recorded, as accurately as possible in terms of quality and quantity, the food and beverages consumed during two working days and one weekend or public holiday. These data were processed and analysed using Dietowin software (version 7.1, Barcelona, Spain).
- FFQ was administered to determine the frequency of consumption of different food groups. To assess the adequacy of nutrient and food group consumption, the recommendations and reference values established by the European Food Safety Authority (EFSA) [27], the Spanish Food Safety and Nutrition Agency (AESAN) [28], and the Spanish Society of Community Nutrition (SENC) were taken as a reference [29].

#### 2.3.2. The Speech–Language Assessment

Two validated screening protocols were applied to detect dysphagia: the Yale Swallow Protocol (YSP) [30] and the Test of Mastication and Swallowing Solids (TOMASS) [31].

- The YSP allows for the identification of a possible risk of dysphagia with liquids through a combination of cognitive assessment, examination of the orofacial mechanisms involved in swallowing, and a water intake test, consisting of swallowing a 90 mL glass of water. The appearance of warning signs, such as coughing or a wet voice, is considered indicative of a risk of dysphagia [30].
- The TOMASS test assesses solid swallowing by ingesting a standardised portion of biscuit, while the researcher records temporal and quantitative parameters, such as total swallowing time, number of chews and swallows, the need for forced swallows, and the presence of warning signs like those observed in the YSP [31].

During the pre-test assessment, specific questions were included to identify whether participants perceived any changes in saliva production (either decreased or increased) and whether they had difficulty eating foods that combine two or more textures, such as certain fruits (e.g., oranges). Both tools provide clinical screening information and do not constitute instrumental confirmation of swallowing impairment (e.g., FEES or video fluoroscopy). All questionnaires and interview-based assessments were administered face-to-face by the researcher to ensure accurate and reliable data collection.

### 2.4. Procedures

With the collaboration of the Hospital Pharmacy Service of the Virgen de las Nieves University Hospital and the GAEN Association, many people diagnosed with MS were informed about the study. The objectives and design of the project were explained, including its duration (six months), the need for frequent meetings, and the degree of participation required. Following this information process, 30 participants were selected and recruited. During the first visit, the procedure was reiterated, the voluntary nature of participation was emphasised, and compliance with the inclusion criteria was verified. Informative documentation was provided, and the informed consent form was signed. The initial and final assessments followed a similar structure and lasted approximately 60 min each. The order of the tests was: (1) anthropometric and body composition assessment, (2) speech therapy assessment, and (3) nutritional status assessment. All measurements were performed following standardised protocols to ensure comparability between the two assessments.

### 2.5. Data Analysis

Statistical processing and analysis were performed using IBM SPSS Statistics 23.0 (Statistical Package for the Social Sciences Inc.; Armonk, NY, USA). Given that each subject was assessed at two points in time, the analysis focused on comparing paired measures to determine both concordance with reference parameters and the presence of temporal changes. For convenience, the first assessment was designated t0 and the second t2. The distribution of continuous variables was checked using the Shapiro–Wilk test and visual inspection of histograms, and a non-parametric approach was adopted. The Wilcoxon test for related samples was used for comparisons between the two assessments of continuous variables, and the McNemar test was applied for dichotomous qualitative variables. The threshold for statistical significance was set at *p* < 0.05, and the results were accompanied by 95% confidence intervals. Qualitative variables are presented as frequencies and percentages, while quantitative variables are summarised using median and interquartile range (P25–P75) to reflect the non-parametric distribution of the data. The temporal change in variables was calculated using the difference between measurements at t2 and t0 (Δ = t2 − t0). Additionally, exploratory correlation analyses were conducted between dysphagia-related variables and nutritional indicators; however, none of these correlations reached statistical significance.

## 3. Results

The general characteristics of the sample are summarised in Table 1. The cohort consisted of 30 participants, predominantly women, with RRMS as the most frequent clinical phenotype (90%). The median age was 53.3 years at baseline assessment (t0) and 54.0 years at the final assessment (t2). No statistically significant differences were detected between the variables compared at t0 and t2. Elevated BMI values (BMI ≥ 25) were observed, together with an increase in the proportion of participants classified as overweight, rising from 43.3% at t0 to 66.7% at t2.

The findings derived from the speech–language pathology assessment are presented in Table 2. At t0, salivary alterations were observed by 50% of participants, and the overall risk of dysphagia was 63.3%. At t2, the prevalence of facial tension (up to 66.7%) and the risk of dysphagia (76.7%) increased. No statistically significant differences were detected between the two assessments in most of the parameters analysed, except for facial tension, which showed a significant increase, rising from 36.7% at t0 to 66.7% at t2 (*p* = 0.020). The comparison between assessments showed that 40% of participants changed from not presenting facial tension to presenting it. In summary, more than half of the study sample showed indications of dysphagia risk in at least one of the two assessments, with a greater presence of signs compatible with dysphagia to liquids at both evaluation time points.

Table 3 summarizes the adequacy of macro- and micronutrient intake in the sample. In both evaluations, a high prevalence of inadequate nutrient intake was observed. At t0, more than half of the sample showed inadequate intake of energy (70.0%), carbohydrates (53.3%), fibre (80.0%), calcium (53.3%), iron (53.3%), potassium (80.0%), and magnesium (70.0%). At t2, a similar pattern observed for energy (70.0%), fibre (70.0%), sodium (66.7%), potassium (66.7%), and magnesium (60.0%), with statistically significant differences between both time points for sodium inadequacy (*p* = 0.02) and iron (*p* = 0.02). These variations reflect both improvements and deteriorations in dietary quality; for example, the number of participants with inadequate iron intake decreased from 16 to 9 subjects, whereas inadequate sodium intake increased from 11 to 20 subjects.

Table 4 presents the frequency of food consumption of the different food groups among the study participants. At t0, it was observed that more than half of the sample exhibited inadequate intake across all food groups. However, water and vegetable consumption constituted an exception, as 30% and 16.7% of participants, respectively, showed values below the established recommendations. At t2, a partial improvement in dietary patterns was observed, although the proportion of inadequacy remained high in several food groups; specifically, for water, vegetables, nuts, and eggs, inadequacy was observed in less than 50% of the sample. When statistically comparing t0 and t2, no significant differences were found, except for olive oil consumption (*p* = 0.008), which showed a significant improvement in intake (according to recommendations), although it did not reach the recommended frequency of consumption.

## 4. Discussion

In the present study, the sample consisted of 30 participants (20 women), with a predominance of RRMS (90%), a median age of 53–54 years, and a median disease duration of 18 years. A concerning combination of orofacial alterations and persistent nutritional deficits was observed. The risk of dysphagia was high at baseline and increased after six months follow-up, accompanied by a significant increase of facial tension. As detailed below, dysphagia to liquids was more frequent than dysphagia to solids at both time points. In the nutritional domain, insufficient intakes of energy, fibre, and several micronutrients—calcium, iron, potassium, magnesium—predominated, along with a high prevalence of overweight that increased from 43.3% to 66.7%. Among the relevant variations observed throughout the study, improvements in food patterns were noted, according to requirements, in iron intake and in the consumption of nuts, eggs, and olive oil, in contrast to an increase in inadequate sodium intake.

The general characteristics of the sample indicate a higher prevalence of MS in women (2:1), as well as a predominance of an RRMS diagnosis (90%). These sociodemographic data are consistent with those reported in other recent studies [2,32,33]. Regarding sex distribution, several studies conducted in Spain report a female-to-male ratio close to 2:1 [34,35]. Likewise, the most frequent clinical form according to the literature is RRMS [5,35,36], a pattern that is also observed in our study. The median disease duration was 18 years, a figure very similar to that reported in a study conducted in Asturias, Spain [35], where the median was 17 years.

Regarding the anthropometrical profile, our study highlights a BMI above the range considered normal (25.5 kg/m^2^), which is consistent with other studies conducted in the MS population [37,38]. In their systematic review, Pilutti et al. [37] analysed 37 studies comprising a total of 2127 individuals with MS, and reported that in those using BIA, BMI values ranged from 21.2 to 25 kg/m^2^, with values closer to the upper limit being more frequent. Given the wide heterogeneity among studies, methodologies, and participant characteristics, it is not surprising to find studies reporting even higher values. For example, Venasse et al. [38], in another trial involving 60 participants with MS, reported a BMI of 25.8 kg/m^2^. Although no significant differences were found over time, it is noteworthy that at the second evaluation an increase in overweight was observed in the sample, which rose from 43.3% to 66.7%.

Several aspects were identified in speech–language pathology assessment. The risk of dysphagia, which was already high at the first evaluation (63.3%), increased at the second, reaching 76.7% of the sample. Both at the initial and final assessments, a high prevalence of dysphagia risk was observed among participants. As reported in other studies [6], dysphagia may manifest in the early stages of the disease and becomes more frequent as MS progresses, which could explain the increase observed in our participants. The literature includes numerous studies and systematic reviews attempting to establish its prevalence; however, this task is complex due to the substantial variability associated with the assessment methods used. Some studies describe wide ranges, from 38% to 81% [23], whereas others have estimated a pooled prevalence of approximately 45.3% [39].

In our study, a higher frequency of dysphagia to liquids than solids was observed at both evaluations. Although the most recent literature is somewhat limited, this pattern has also been described in early research [40]. Depending on the evaluation time point, two parameters were identified that could influence the presence of dysphagia: at the first assessment, salivary alteration—primarily manifested as oral dryness—was prominent, whereas at the second assessment, an increase in facial tension was observed. Xerostomia and reduced salivary flow are relevant risk factors, as saliva not only lubricates but also performs essential physiological functions for swallowing. These include facilitating food transit, enabling adequate bolus formation, reducing the adherence of residues in the oral cavity, stimulating the swallowing reflex, and participating in the initiation of nutrient digestion [41]. Regarding facial muscle tension, which is often associated with temporomandibular dysfunction, it can cause overload or spasm of the masseter muscle (due to stress, bruxism, or poor posture), resulting in facial pain, stiffness, and limitations in mandibular mobility. These alterations directly impact mastication and may necessitate dietary modifications, favouring the consumption of soft-textured foods [41]. As reported by previous studies [11,23,39,42], we consider that dysphagia in MS remains underdiagnosed, being poorly evaluated and recognized by both patients and healthcare professionals. In our sample, only 16.7% had previously consulted a speech–language pathologist, and in some cases, the role of this professional was even unknown. Other studies support this perspective and emphasize the importance of the speech–language pathologist in the care of these patients [6].

Another focus of this research was to characterize the nutritional profile of the MS population. Defining an optimal nutritional pattern for individuals with MS is a considerable challenge, largely due to the multifactorial nature of the disease. It should be noted that MS is a chronic condition with neurodegenerative, autoimmune, and inflammatory components, and adequately addressing the nutritional needs associated with each of these dimensions is a complex task. The available literature shows considerable heterogeneity, including studies exploring specific dietary patterns [14,16,17,43,44], and investigations focused on the role of certain macro- and micronutrients in disease progression [45,46], as well as studies centred on the use of complementary nutritional supplementation [18,47,48]. Identifying studies focused on macro- and micronutrient intake, food group consumption, or, more specifically, on the Spanish population with a typically Mediterranean dietary pattern, has not been straightforward. The available evidence is limited: few studies analyse overall dietary intake and the nutritional characteristics of this population [15,38,49].

Focusing on the obtained results, in general terms, both in the assessment of macronutrients and micronutrients, a high proportion of inadequacy was observed. The only nutrient that showed a statistically significant difference between the initial evaluation and the follow-up evaluation was iron, whose intake increased, reducing inadequacy from 53.3% to 30% of the sample. For the remaining nutrients, no significant changes were recorded, and overall, most showed high rates of inadequacy. Although slight reductions in inadequacy were observed for some parameters (e.g., iron, carbohydrates, fibre, and potassium), other variables worsened (e.g., sodium and protein). These findings can be compared with those of Afifi et al. [44], who evaluated dietary intake and eating habits in an MS population before and after a 3-month intervention: in comparison, our sample presented lower absolute intakes, especially for energy, and, as in Afifi et al. [44], no significant overall changes were observed between measurements. The differences found between studies may be explained by the Mediterranean dietary pattern, cultural habits, and/or recording methods.

Regarding consumption frequency, the findings indicate that dietary inadequacies were common at baseline, particularly across several key food groups. Although a partial improvement was observed after 6 months, most inadequacies persisted, suggesting only modest and selective short-term changes. Overall, these findings indicate modest and selective short-term dietary changes, which should be interpreted cautiously given the study’s observational design and sample size; the present data do not allow us to conclude that more intensive or sustained interventions would necessarily correct these inadequacies. Among foods with potentially beneficial effects in MS, vegetables and fruits, fish, nuts, and extra virgin olive oil stand out, as they provide a wide range of nutrients and compounds with possible neuroprotective, immunomodulatory, antioxidant, and anti-inflammatory effects [14,48,50], making it advisable to promote the consumption of these foods. In addition to dietary patterns, nutritional supplementation has also been explored as a complementary strategy in MS. Although the evidence remains heterogeneous [47], certain micronutrients, such as vitamin D, omega-3 fatty acids, biotin in specific contexts, and selected antioxidants [16,47,51,52], have shown potential effects on inflammation, oxidative stress, and immune function. Nevertheless, the benefits tend to be modest and depend on dosage, duration, and the patient’s baseline status, meaning that supplementation should be considered as supportive rather than a substitute for sustained dietary interventions or disease-modifying therapy.

Beyond supplementation, it is also important to consider the broader interaction between nutrition and clinical manifestations relevant to MS. Although the number of studies examining the relationship between dysphagia and nutrition in MS has increased in recent years [53], this remains an emerging field, and the literature often addresses both aspects separately. In the speech and language therapy domain, for example, the meta-analysis by Chu et al. [53] shows that texture modified diets in individuals with dysphagia are associated with improved intake and reduced aspiration episodes. From a nutritional perspective, numerous studies [14,15,16] have explored dietary approaches in MS; however, they do not conclusively identify the most appropriate diet for this population, largely due to sample heterogeneity. Even so, several works agree that diet can modulate inflammatory and oxidative processes involved in MS pathophysiology, providing a strong rationale for considering nutrition as a relevant component of clinical care. According to the previous literature, appropriate nutritional intervention may help reduce fatigue, improve quality of life, decrease inflammatory markers, promote neuroprotection, and optimise metabolic parameters associated with disease progression. Dietary patterns with an anti-inflammatory profile, such as the Mediterranean diet, low saturated fat diets, or those rich in antioxidants, have shown benefits in symptoms, wellbeing, and metabolic control. Although clinical evidence remains heterogeneous, the available data support the role of nutrition as a complementary and potentially beneficial strategy within the comprehensive management of people with MS.

Finally, one of the objectives of the study was to observe whether the evaluated parameters change over a 6-month assessment period. No statistically significant differences were detected between time points; this result should be interpreted with caution, as it indicates that, under the conditions and with the sample studied, there was no support to reject the null hypothesis, but it does not rule out the existence of small effects or effects dependent on other conditions. A larger sample size and a longer interval between evaluations could increase the likelihood of detecting additional outcomes.

This study presents several strengths, such as the use of validated instruments administered face-to-face by trained research staff, which enhances the reliability of the data obtained. However, some limitations should also be acknowledged. The sample size was small, and participant retention during the follow-up period was challenging, which may have limited the ability to detect small effects. Likewise, the six-months interval between assessments may have been insufficient to identify more subtle longitudinal changes. In addition, the dietary assessment methods used in this study (72 h dietary recall and the FFQ) are subject to inherent limitations. Both tools rely on participants’ memory and self-reporting, which may introduce recall bias and misreporting, particularly in populations with cognitive fatigue or fluctuating daily routines, such as individuals with MS. These factors may affect the accuracy of the estimated energy and nutrient intakes. Although these methods are widely used in nutritional epidemiology and offer practical advantages, their potential for under- or over-estimation should be considered when interpreting the findings. Additionally, certain clinical variables that could act as potential confounders—such as EDSS, detailed pharmacological treatment, and objective measures of physical activity—were not available for all participants, which further limits the interpretability of the findings. Moreover, the observational design precludes causal inference, and therefore clinical recommendations derived from the findings should be interpreted with caution.

## 5. Conclusions

This study revealed a high prevalence of dysphagia risk in patients with MS, along with elevated BMI and inadequacies in macro- and micronutrient intake, as well as inadequate consumption of certain food groups relevant to disease progression. No significant differences were detected between the initial and final evaluations.

Although the observational design does not allow causal inferences, the coexistence of swallowing difficulties and suboptimal nutritional patterns observed in this cohort highlights the importance of further research exploring both aspects jointly. Our findings do not support specific clinical recommendations, but they suggest that these domains may warrant attention in future studies. The need for future research with larger sample sizes and long-term follow-up is emphasized, to more accurately clarify the relationship between swallowing disorders and nutritional status in individuals with MS.

## Figures and Tables

**Table 1:** Main descriptive and anthropometric characteristics of adults with MS.

	First Evaluation (t0) MS Patients (*n* = 30)	Second Evaluation (t2) MS Patients (*n* = 30)	Pre–Post Difference Δ (t_2_ − t_0_): Worsened (Δ < 0)	Statistic
Type of MS (*n*, %)			-	
RRMS	27 (90.0%)			
SPMS	2 (6.7%)			
PPMS	1 (3.3%)			
Sex (*n*, %)			-	
Male	10 (33.3%)			
Female	20 (66.7%)			
Duration MS (years)	18 (13.5–23.8)		-	
Age (years)	53.5 (45.3–59.8)	54 (46.3–60.0)	-	Z = −4.583; *p* = 0.000
Height (m)	1.67 (1.60–1.71)		-	
Weight (kg)	71.5 (64.3–78.8)	69.9 (64.0–82.5)	-	Z = −1.004; *p* = 0.316
BMI (kg/m^2^)	25.5 (24.2–28.7)	25.8 (23.8–29.5)	-	Z = −1.091; *p* = 0.275
BMI status (*n*, %)			4 (13.3%)	
Underweight (BMI < 18.5)	1 (3.3%)	1 (3.3%)		
Normal weight (BMI 18.5–24.9)	13 (43.3%)	9 (30.0%)		
Overweight (BMI ≥ 25)	16 (53.3%)	20 (66.7%)		
FM (%)	28.8 (22.7–34.0)	30.1 (24.5–33.9)	-	Z = −0.761; *p* = 0.447
FFM (kg)	49.5 (45.5–56.7)	50.7 (45.0–56.8)	-	Z = −0.195; *p* = 0.846
Hydration (type, %)			2 (6.7%)	McNemar: b = 2, c = 4; *p* = 0.687
Normo-hydrated	28 (93.3%)	26 (86.7%)		
Dehydrated or overhydrated	2 (6.7%)	4 (13.4%)		
Nutrition (type, %)			1 (3.3%)	McNemar: b = 0, c = 1; *p* = 1.000
Normal values	29 (96.7%)	28 (93.3%)		
Malnourished	1 (3.3%)	2 (6.7%)		
BMR (kcal/day)	1495 (1421–1650)	1495 (1443–1675)	-	Z = −0.573; *p* = 0.567

Note: BMI reference values were extracted from the World Health Organization (WHO). Data are expressed as median and interquartile range (25th–75th percentiles) for continuous variables, and as *n* (%) for categorical variables. Pre–post difference (Δ): cases of deterioration were recorded. Statistics: Wilcoxon signed-rank test (W) was used for paired continuous/ordinal comparisons; McNemar test was used for paired dichotomous comparisons (discordant counts b and c or exact *p* reported when appropriate); *p*-value: two-tailed significance level; differences considered significant at *p* < 0.05. Abbreviations: MS: multiple sclerosis; BMI, Body Mass Index; FM, fat mass; FFM, fat-free mass; BMR, basal metabolic rate; RRMS: relapsing remitting multiple sclerosis; SPMS: secondary progressive multiple sclerosis; PPMS: primary progressive multiple sclerosis.

**Table 2:** Speech–language aspects of adults with MS.

	First Evaluation (t0) MS Patients (*n* = 30)	Second Evaluation (t2) MS Patients (*n* = 30)	Pre–Post Difference Δ (t_2_ − t_0_): Worsened (Δ < 0)	Statistic (*p*-Value)
Attends speech therapy (Yes) (*n*, %)	5 (16.7%)	5 (16.7%)	0 (0%)	0.000 (1.000)
Smokes (Yes) (*n*, %)	7 (23.3%)	7 (23.3%)	0 (0%)	0.000 (1.000)
Facial muscle tension (Yes) (*n*, %)	11 (36.7%)	20 (66.7%)	12 (40%)	−2.324 (0.020)
Facial muscle pain (Yes) (*n*, %)	7 (23.3%)	4 (13.3%)	1 (3.3%)	−1.342 (0.180)
Bruxism (Yes) (*n*, %)	9 (30.0%)	7 (23.3%)	1 (3.3%)	−1.000 (0.317)
Double texture alert (Yes) (*n*, %)	8 (26.7%)	9 (30.0%)	1 (3.3%)	−1.414 (0.157)
Salivation alteration			6 (20%)	−0.905 (0.366)
(Yes) (*n*, %)				
Total	15 (50%)	12 (40%)		
Excess	1 (3.3%)	3 (10.0%)		
Absence	14 (46.7%)	9 (30.0%)		
Risk of dysphagia			6 (20%)	−1.414 (0.157)
(Yes) (*n*, %)				
Total	19 (63.3%)	23 (76.7%)		
Risk of dysphagia to liquid	10 (33.3%)	12 (40.0%)		
Risk of dysphagia to solid	3 (10.0%)	4 (13.3%)		
Risk of mixed dysphagia	6 (20.0%)	7 (23.3%)		

Note: In the variable “Alteration of salivation”, “Excess” indicates hypersalivation and “Absence” indicates hyposalivation. “Double texture alert” refers to a defensive response such as coughing or bolus fragmentation in response to abrupt changes in food consistency, for example soups versus oranges. Data are expressed as *n* (%) for categorical variables. Pre–post difference (Δ): cases of deterioration were recorded. Statistics: Wilcoxon signed-rank test (W) was used for paired continuous/ordinal comparisons; McNemar test was used for paired dichotomous comparisons (discordant counts b and c or exact *p* reported when appropriate); *p*-value: two-tailed significance level; a difference is considered significant when *p* < 0.05. Abbreviations: MS, multiple sclerosis.

**Table 3:** Analysis of daily intake and adequacy of macronutrients and micronutrients in adults with MS.

	First Evaluation (t0) MS Patients (*n* = 30)		Second Evaluation (t2) MS Patients (*n* = 30)		Pre–Post Difference Δ (t_2_ − t_0_): Worsened (Δ < 0)	Statistic W (*p*-Value)
	Median (IQR)	Insufficiency and excess (*n*, %)	Median (IQR)	Insufficiency and excess (*n*, %)		
Energy (kcal)	1484 (1179–1838)	21 (70.0%)	1571 (1304–1915)	21 (70.0%)	4 (13.3%)	0.000 (1.000)
Carbohydrates (%E)	45.4 (39.6–50.7)	16 (53.3%)	47.4 (43.9–54.9)	12 (40.0%)	3 (10%)	−1.265 (0.206)
Protein (%E)	18.1 (16.4–19.8)	8 (26.7%)	19.0 (17.6–23.2)	13 (43.3%)	8 (26.7%)	−1.508 (0.132)
Total fat (%E)	33.4 (29.7–39.7)	13 (43.3%)	31.8 (29.5–36.1)	12 (40.0%)	4 (13.3%)	−0.333 (0.739)
Fibre (g/day)	16.5 (12.5–21.9)	24 (80.0%)	15.9 (13.6–24.4)	21 (70.0%)	5 (16.7%)	−0.832 (0.405)
Calcium (mg/day)	787 (535–850)	16 (53.3%)	759 (624–956)	14 (46.7%)	3 (10%)	0.000 (1.000)
Sodium (mg/day)	1606 (1324–1944)	11 (36.7%)	1826 (1404–2407)	20 (66.7%)	12 (40%)	−2.324 (0.020)
Iron (mg/day)	11.1 (8.93–14.8)	16 (53.3%)	12.7 (9.20–15.6)	9 (30.0%)	1 (3.3%)	−2.333 (0.020)
Phosphorus (mg/day)	1042 (816–1336)	-	1147 (1021–1319)	-	-	0.000 (1.000)
Potassium (mg/day)	2250 (1707–2549)	24 (80.0%)	2598 (2234–3074)	20 (66.7%)	2 (6.7%)	1.414 (0.157)
Magnesium (mg/day)	215 (182–266)	21 (70.0%)	239 (204–306)	18 (60.0%)	5 (16.7%)	0.832 (0.405)

Note: Continuous variables are expressed as median and interquartile range (25th–75th percentiles). Categorical variables are expressed as number and percentage of subjects (*n* (%)). In the absence of defined reference intervals, intake inadequacy was defined as intake < 80% (insufficiency) or > 120% (excess) of the reference; adequate intake was considered between 80% and 120%. Energy requirements for each patient were calculated using the Mifflin–St Jeor equation with a physical activity level (PAL) factor of 1.4, corresponding to light physical activity. Reference values for macro- and micronutrient intakes were taken from the European Food Safety Authority (EFSA) and the Spanish Agency for Food Safety and Nutrition (AESAN). Pre–post difference (Δ): cases of deterioration were recorded. Statistic: Wilcoxon signed-rank test (W) was used for paired continuous/ordinal comparisons to assess changes in dietary intake adequacy; *p*-value: two-tailed significance level; a difference is considered significant when *p* < 0.05. Abbreviation: MS, multiple sclerosis; %E, percentage of total energy.

**Table 4:** Frequency and adequacy of food consumption in adults with MS.

	RF	First Evaluation (t0) MS Patients (*n* = 30)		Second Evaluation (t2) MS Patients (*n* = 30)		Pre–Post Difference Δ (t_2_ − t_0_): Worsened (Δ < 0)	Statistic W (*p*-Value)
		Median (IQR)	Insufficiency and excess (*n*, %)	Median (IQR)	Insufficiency and excess (*n*, %)		
Water	4–8 s/d	5.00 (4.00–8.00)	9 (30%)	5.00 (4.00–8.00)	8 (26.7%)	4 (13.3%)	−0.333 (0.739)
Bread, cereals, rice, pasta and potatoes	4–6 s/d	1.86 (1.45–2.69)	27 (90%)	2.21 (1.35–2.54)	29 (96.7%)	2 (6.7%)	−1.414 (0.157)
Milk and dairy products	2–4 s/d	2.07 (1.38–3.53)	19 (63.3%)	2.11 (1.33–3.57)	18 (60.0%)	5 (16.7%)	−0.302 (0.763)
Vegetables and greens	≥2 s/d	4.28 (2.59–5.25)	5 (16.7%)	3.42 (1.96–5.37)	8 (26.7%)	4 (13.3%)	−1.342 (0.180)
Fruit	≥3 s/d	1.56 (0.76–2.87)	22 (73.3%)	1.64 (0.97–2.72)	23 (76.7%)	5 (16.7%)	−0.333 (0.739)
Olive oil	3–6 s/d	1.00 (1.00–2.00)	30 (100.0%)	2.00 (1.00–2.46)	23 (76.7%)	0 (0%)	−2.646 (0.008)
Legumes	2–4 s/w	2.51 (1.17–4.00)	18 (60.0%)	2.47 (0.98–4.52)	19 (63.3%)	5 (16.7%)	−0.333 (0.739)
Nuts	3–7 s/w	1.50 (0.92–7.00)	17 (56.7%)	3.50 (0.64–7.00)	12 (40.0%)	2 (6.7%)	−1.667 (0.096)
Fish and seafood	3–4 s/w	4.74 (3.67–6.74)	26 (86.7%)	6.35 (4.02–7.96)	28 (93.3%)	2 (6.7%)	−1.414 (0.157)
Lean meats, poultry	3–4 s/w	4.85 (3.00–8.25)	23 (76.7%)	3.18 (2.00–5.00)	18 (60.0%)	4 (13.3%)	−1.387 (0.166)
Eggs	3–4 s/w	3.00 (2.00–4.12)	19 (63.3%)	3.00 (3.00–4.25)	13 (43.3%)	6 (20.0%)	−1.414 (0.157)
Processed meats and fatty meats ^a^	o/m	4.50 (2.92–6.77)		4.23 (3.00–6.85)			
Sweets, snacks, soft drinks ^a^	o/m	7.63 (4.20–11.24)		9.23 (7.00–21.00)			
Margarine, butter, pastries ^a^	o/m	5.08 (2.71–9.07)		7.21 (2.00–9.29)			
Wine/beer ^a^	o/m	1.00 (0.00–3.42)		0.47 (0.00–2.54)			

Note: Continuous variables are expressed as median and interquartile range (25th–75th percentiles). Categorical variables are expressed as number and percentage of subjects. Reference values for recommended consumption frequencies were taken from the Spanish Society of Community Nutrition (SENC). ^a^ When occasional rations are recommended or when no specific recommendation exists for a food group, intake is reported as servings/week. Pre–post difference (Δ): cases of deterioration were recorded. Statistic: Wilcoxon signed-rank test (W) was used for paired continuous/ordinal comparisons to assess changes in dietary intake adequacy; *p*-value: two-tailed significance level; a difference is considered significant when *p* < 0.05. Abbreviations: MS, multiple sclerosis; RF, recommended frequencies; IE, Insufficiency and excess; s/d, servings/day; s/w, servings/week; o/m, Occasional/moderate.

## Data Availability

Data is available on request due to restrictions on privacy. The data presented in this study are available on request from the corresponding author.

## Abbreviations

The following abbreviations are used in this manuscript:

MS	Multiple Sclerosis
BMI	Body mass index
GAEN	Association of People Affected by Neurodegenerative Diseases of Granada
FFQ	Food frequency questionnaire
BIA	Bioelectrical Impedance Analysis
FM	Fat mass
FFM	Fat-free mass
BMR	Basal metabolic rate
EFSA	European Food Safety Authority
AESAN	Spanish Food Safety and Nutrition Agency
SENC	Spanish Society of Community Nutrition
YSP	Yale Swallow Protocol
TOMASS	Test of Mastication and Swallowing Solids
RRMS	Relapsing–remitting multiple sclerosis
SPMS	Secondary progressive multiple sclerosis
PPMS	Primary progressive multiple sclerosis
